# Long-Term Performance of Monolithic Silica Aerogel with Different Hydrophobicities: Physical and Color Rendering Properties after an Accelerated Aging Process

**DOI:** 10.3390/gels9030210

**Published:** 2023-03-10

**Authors:** Costanza Vittoria Fiorini, Francesca Merli, Elisa Belloni, Mary K. Carroll, Ann M. Anderson, Cinzia Buratti

**Affiliations:** 1 DIAEE Department of Astronautical, Electrical, and Energy Engineering, “Sapienza” University of Rome, Via Eudossiana 18, 00184 Rome, Italy; 2 Department of Engineering, University of Perugia, Via G. Duranti 63, 06125 Perugia, Italy; 3 Chemistry Department, Union College, 807 Union Street, Schenectady, NY 12308, USA; 4 Mechanical Engineering Department, Union College, 807 Union Street, Schenectady, NY 12308, USA

**Keywords:** monolithic aerogel, hydrophobicity, accelerated aging, optical properties, acoustic performance, color rendering index

## Abstract

Due to its excellent properties, monolithic silica aerogel is a promising material for innovative glazing systems. Since glazing systems are exposed to deteriorating agents during building service life, it is essential to investigate the long-term performance of aerogel. In the present paper, several 12.7 mm-thick silica aerogel monoliths produced by a rapid supercritical extraction method were tested, including both hydrophilic and hydrophobic samples. After fabrication and characterization of hydrophobicity, porosity, optical and acoustic properties, and color rendering, the samples were artificially aged by combining temperature and solar radiation effects in an experimental device specifically developed at the University of Perugia. The length of the experimental campaign was determined using acceleration factors (AFs). Temperature AF was evaluated according to the Arrhenius law using thermogravimetric analysis to estimate the aerogel activation energy. A natural service life of 12 years was achieved in about 4 months, and the samples’ properties were retested. Contact angle tests supported by FT-IR analysis showed loss of hydrophobicity after aging. Visible transmittance values in the 0.67–0.37 range were obtained for hydrophilic and hydrophobic samples, respectively. The aging process involved optical parameter reduction of only 0.02–0.05. There was also a slight loss in acoustic performance (noise reduction coefficient (NRC) = 0.21–0.25 before aging and NRC = 0.18–0.22 after aging). For hydrophobic panes, color shift values in the 10.2–59.1 and 8.4–60.7 ranges were obtained before and after aging, respectively. The presence of aerogel, regardless of hydrophobicity, results in a deterioration in light-green and azure tones. Hydrophobic samples had lower color rendering performance than hydrophilic aerogel, but this did not worsen after the aging process. This paper makes a significant contribution to the progressive deterioration assessment of aerogel monoliths for applications in sustainable buildings.

## 1. Introduction

Building materials are subjected to environmental and atmospheric agents throughout their service life, so understanding their long-term behavior is crucial. Many factors, such as wind, rain, solar radiation, temperature variation, and humidity, influence their performance. Accelerated aging tests can be carried out at a laboratory scale by subjecting materials to extreme temperature, humidity, and solar radiation conditions that speed up the normal degradation processes. However, evaluating long-term properties is sometimes difficult for innovative materials because no standard methods are available in the literature.

The long-term behavior of aerogel, an innovative building material, is not well understood. Aerogel emerged in the building sector in the 1980s because of its high insulation properties and high transparency, which make it useful for both opaque and transparent envelope applications [1,2,3]. It is available in both granular and monolithic forms. The latter form has attracted research interest in recent years [4,5,6,7,8,9] due to its excellent thermal, optical, and acoustic properties, despite the high cost of production [10].

The effect of aging due to moisture and solar radiation was studied in [4,11] for glazing units with large and small aerogel granules. To simulate an actual aging period of 5 years, the samples were subjected to a 35–65 °C temperature range and relative humidity (RH) of 100% for 300 cycles of about 6 h for a total period of 3 months. Negligible variation in the aerogel moisture content was measured due to the hydrophobicity of the material, whereas the thermal conductivity increased by 5–10%. To simulate an aging period of 12.5 years, a 1200 W/m^2^ lamp was used to irradiate the samples during the same 300 cycles; a slight reduction in hydrophobicity was observed, and the solar irradiation caused a minimal decrease in the contact angle. On the other hand, other studies on granular aerogel showed no significant effects on thermal conductivity when samples were aged, according to the results of IEA-EBC Annex 65 “Long-Term Performance of Super-Insulating-Materials (SIM) in Building Components and Systems” [12,13]. A slight increase in thermal conductivity was observed over time caused by increased moisture content inside the insulating material.

Concerning opaque envelope applications, Berardi and Nosrati [14] studied different aerogel-based materials (plasters, blankets, and boards) subjected to an accelerated laboratory aging process to investigate the effect of up to 20 years of aging. Among the weathering agents studied, high relative humidity had the most significant impact on thermal performance due to the residual moisture in the material. For 70% vol. aerogel-based plaster, the thermal conductivity increased by 10% over time, reaching a value of 0.035 W/mK.

No studies are available in the literature on the long-term performance of monolithic aerogel. At Union College (Schenectady, New York, United States), a rapid supercritical extraction technique was developed to manufacture monolithic aerogel samples: this method can reduce the time of fabrication and reagent waste [15,16,17]. Monolithic aerogel systems can yield high transparency when installed in glazing applications: thermal and acoustic insulation characteristics are relevant, as well as the optical and color rendering properties and the light quality. The lighting quality of aerogel glazing was analyzed in terms of diffuse transmittance, and a behavior similar to standard glazing units was observed [18]. In order to assess the quality of lighting for a monolithic aerogel pane sandwiched between two glazing panes, Zinzi et al. investigated the visual distortion of objects observed through a sample [10], and results showed that the visual distortion became high only when very small objects were observed. Color rendering is also an important feature to consider: a new methodology was developed to evaluate the effect of an aerogel pane put over a reference color palette to estimate the color rendering index (R_a_), as reported in [19]. Results showed that monolithic aerogel tends to make the colors brighter, and in general, the color shift decreased when decreasing the percentage ratios of the blue coordinate.

All these properties could be analyzed for different kinds of samples both before and after an accelerated aging period. The difficulty is finding a configuration for the accelerated aging method for aerogel. In particular, activation energy strongly affects the temperature’s acceleration factor (AF) value. Generally, there is a tendency to assume mean values of acceleration factors that can be considered valid for most standard building materials. However, no activation energy values for aerogel monoliths were found in the literature. In previous research, an experimental campaign with thermogravimetric analysis was carried out to address this issue with both granular and monolithic aerogel [20]. A reliable value of the activation energy was determined for use with the Arrhenius law to calculate the acceleration factor for temperature.

In the present paper, several 12.7 mm-thick silica aerogel samples produced by a rapid supercritical extraction method were tested in both hydrophilic and hydrophobic forms. The samples were aged by combining temperature and solar radiation effects in an innovative experimental device developed at the University of Perugia. A service life equivalent to about 12 years was achieved in 4 months (determined by the acceleration factor (AF) found in [20]). Properties were evaluated before and after aging and include: spectral transmittance and reflectance, acoustic absorption coefficients, acoustic insulation properties, color rendering [19], density, surface area, porosity and contact angle. The influence of the samples’ hydrophobicity on the material’s performance before and after aging was also investigated.

This paper presents the first investigation of the influence of hydrophobicity of monolithic silica aerogel panes on the physical and color rendering properties of the material and contributes to the limited literature on the effects of aging on the long-term performance of aerogel materials.

## 2. Materials and Methods

### 2.1. Description of the Samples

**Figure 1:**
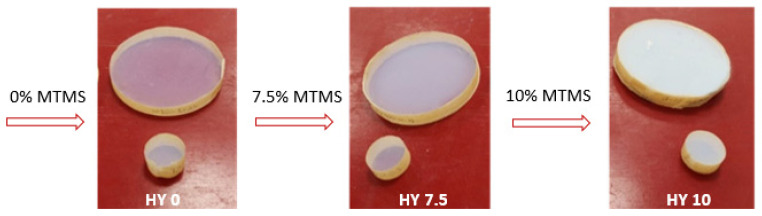
Hydrophilic (HY 0) and hydrophobic (HY 7.5 and HY 10) investigated samples.

Several monolithic cylindrical aerogel samples of 100 mm and 29 mm diameter and 12.7 mm thick (Figure 1) were fabricated employing a rapid supercritical extraction method (RSCE) [15,16,17]. The precursor chemical recipe used to fabricate hydrophilic samples (HY 0) was tetramethyl orthosilicate (TMOS), reagent grade methanol (MeOH), in-house deionized water, and 1.5 M ammonium hydroxide (NH_3_) mixed in a molar ratio of 1:12:3.7:0.007 (TMOS:MeOH:H_2_O:NH_3_). Hydrophobic samples were prepared by replacing 7.5% (HY 7.5) and 10% (HY 10) of the TMOS volume with methyltrimethoxysilane (MTMS).

To process the aerogels using RSCE, the precursor chemical mixture was poured into a steel mold sandwiched between gasket material and placed in a 30-ton hydraulic hot press (Tetrahedron MTP-14). The mold was sealed by applying a force of 180–220 kN and the hot press heated it to 288 °C (rate of 1.1–2.2 °C/min). When this supercritical temperature was reached, the system equilibrated for 55 min. Then, the hot press force was lowered (4.45 kN/min), allowing the supercritical fluid to escape. The hot press cooled the mold back to 32° C at the same initial heating rate. The overall duration of the RSCE was 6.5 h for the 28 mm and 10.5 h for the 100 mm sample fabrication.

### 2.2. Aging Process

A detailed description of the procedure for accelerated aging is included in [20]. In order to correlate the accelerating atmospheric agents with natural service conditions and to estimate the required aging time, factors were calculated for both temperature and radiation influence: the acceleration factor for temperature (AF_T_) and the acceleration factor for radiation (AF_RAD_). The combined acceleration factor AF_c_ was estimated by multiplying AF_T_ and AF_RAD_.

AF_T_ was obtained from the Arrhenius formula [21] as a function of the activation energy E_α_, which was evaluated by thermogravimetric analysis due to the lack of reliable literature data for the monolithic aerogel. Crucible tests were performed for three different heating rates (5, 7, and 10 °C/min) in an air atmosphere from 20 ° to 900 °C. The model-free isoconversional Starink and Ozawa–Flynn–Wall (OFW) methods were applied to the thermal degradation results in the 50–100 °C range, and an activation energy equal to 88 kJ/mol was obtained for hydrophilic aerogel (HY 0) [20]. Lower values were found for the hydrophobic samples (45 kJ/mol for HY 7.5 and 65 kJ/mol for HY 10).

AF_RAD_ was calculated from the total energy of the lamps used for accelerated aging and the energy corresponding to natural outdoor aging. This value was fixed from the solar spectrum to the latitude of the Engineering Department (University of Perugia) on a surface incline of 90°, which corresponds to vertical window walls. The first term of the acceleration factor for radiation was calculated thanks to a specific experimental device equipped with 10 lamps (V-tac 4458 6000 K, power of each lamp equal to 17 W [22]) with a spectrum very similar to the solar radiation spectrum. Inside the device, temperature and radiation were recorded at 10-minute intervals using a Babuc acquisition system with cycles of about five days a week (from Monday at 8 am to Friday at 6 pm), allowing a progressive variation of the aging factors due to seasonal temperature differences and to the decay of light power of the lamps. About four months were needed to achieve a natural service life of 12 years, during which acceleration factors’ average values AF_T_ =17.1 and AF_RAD_ = 2.1 were recorded.

### 2.3. Characterization before and after Aging

Optical, acoustic, color rendering, hydrophobicity, and other physical performance of the samples were investigated before and after the aging process.

Spectral transmittance and reflectance properties were measured in the 300–2500 nm wavelength range using a double-beam spectrophotometer (Shimadzu Solid Spec-3700) at the Laboratory of Environmental Control (Department of Engineering, University of Perugia) [23]. It is equipped with a small integrating sphere (60 mm diameter) and three detectors that cover the range from ultraviolet to near-infrared. Light and direct solar transmittance (τ_v_, τ_e_) and reflectance (ρ_v_, ρ_e_) factors were calculated starting from the measurements, in compliance with EN 410 [24].

Normal incidence absorption coefficient α and transmission loss (TL) were measured through an impedance tube equipped with two (α-value) or four (TL-value) 1/4-inch condenser pressure-field microphones [25,26,27], following transfer function and two-load methods, respectively. Large (100 mm diameter) and small (29 mm) samples were used for the measurements in the 100–1600 Hz and the 400–6400 Hz frequency range, respectively. Measured absorption coefficient values were combined with dedicated software, and the trends were obtained in the 100–6400 Hz range.

The methodology developed in [19] was used to evaluate the color rendering of the object observed through the transparent monolithic aerogel using an Illuminator and a Minolta Chroma Meter CR-200. Reference mosaics of 24 (ColorChecker-24) color patches were used for the color detection, and the color shift ΔE values were calculated for each patch based on experimentally obtained CIE Lab coordinates. Moreover, eight test colors were selected from a mosaic of 140 patches to calculate the general color rendering index (R_a_) following standard EN 410 [24].

The sample density was determined using a dial caliper to measure the height and diameter of each sample (for volume calculation) and massing the sample on an analytical balance. Results were reported as the average density ± one standard deviation.

The surface area and pore distributions of the HY 0, HY 7.5 and HY 10 samples (28 mm diameter; 0, 7.5%, and 10% MTMS) were measured by using Micromeritics ASAP 2020. Samples were prepared by lightly crushing approximately 0.2 g of aerogel material. The aerogel was then degassed at 90 °C for two hours and then at 200 °C for an additional six h. An 82-point gas adsorption analysis was performed using a 5 s equilibration time for partial pressures below 0.9 and a 50 s equilibration time for partial pressures above 0.9 to avoid compression effects in the aerogel at high pressure [28]. BET (Brunauer–Emmett–and Teller method) surface area was determined using six points at partial pressures from 0.05 to 0.30. BJH (Barrett–Joyner–Halenda method) pore distributions were determined from the desorption isotherms. Surface area uncertainty estimates were based on the uncertainty in the BET fit and in the mass measurement of the sample (0.005 g).

Contact angles were measured using a Krüss Drop Shape Analysis System (DSA 100). Images of 2 µL drops of deionized water were analyzed using the Laplace–Young method to obtain contact angles. Ten measurements were made on select samples of the as-prepared hydrophobic aerogel samples (HY 7.5 and HY 10). Data are presented as the average contact angle ± one standard deviation.

Infrared spectra of lightly crushed aerogel samples were collected using a Thermo Scientific Nicolet iS5 FTIR spectrometer with an iD7-ATR attenuated total reflectance attachment. Spectra were recorded at 4 cm^−1^ resolution, and sixteen scans were averaged.

## 3. Results and Discussion

### 3.1. Optical Properties

**Figure 2:**
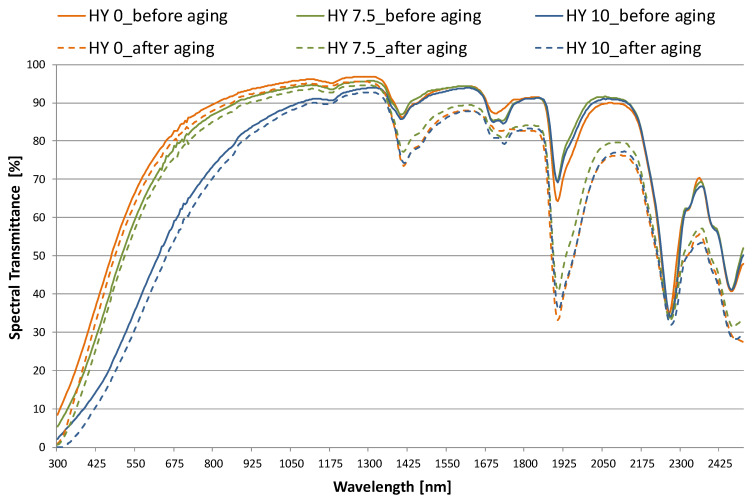
Total transmission coefficient vs. wavelength for monolithic aerogel panes of increasing hydrophobicity: performance before and after 12 years of aging.

**Figure 3:**
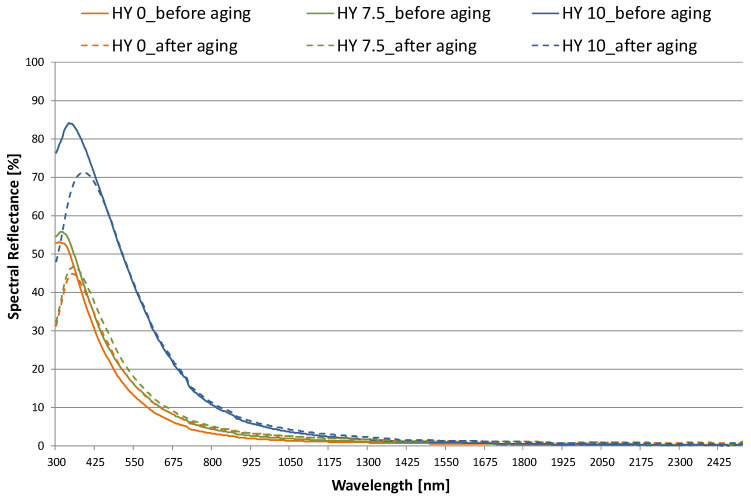
Total reflection coefficient vs. wavelength for monolithic aerogel panes of increasing hydrophobicity: performance before and after 12 years of aging.

Figure 2 and Figure 3 plot total spectral transmittance and reflectance results measured in the 300–2500 nm wavelength range [23] comparing the behavior of as-prepared and aged aerogel samples at each MTMS level (0, 7.5%, and 10%). For each hydrophobicity, at least one 28 mm-diameter and one 100 mm-diameter sample were analyzed. Due to the material inhomogeneity, the measurement on each sample was repeated three or four times by changing its position, and mean values were considered for transmittance and reflectance, respectively. The figures plot the average of the values measured for the large and small samples. The transmission coefficient is high in both the solar and visible spectra. Visible transmittance reaches a maximum value of 0.89 at about 780 nm for the non-hydrophobic aerogel. As expected, spectral transmission decreases as the hydrophobicity of the sample increases. In the visible and near-infrared (NIR) ranges, visible transmittance is reduced up to 4% for HY 7.5 relative to HY 0. Samples with 10% MTMS reveal a further reduction: at 550 nm, τ_HY10_ is equal to 0.35, whereas τ_HY0_ is equal to 0.65. The absorption peaks shifted to higher wavelengths and contribute to the slightly reddened transmitted light when observed through the aerogel; the material displays a slight bluish haze when an illuminated piece is viewed against a dark background. For wavelengths exceeding 1400 nm, hydrophobicity does not significantly influence the transmission coefficient; moreover, selective absorption troughs typical of the material (λ ≈ 1400 nm, λ ≈ 1700 nm, λ ≈ 1900 nm, λ ≈ 2300 nm) are visible in the wavelength range concerned.

After aging, transmittance is slightly reduced across the whole spectrum for all the specimens (Figure 2). The visible maximum falls at 780 nm, and for all three aerogel sample types, it is reduced by a minimum amount, around 2%. Above 1300 nm, the transmission reduction becomes more consistent: characteristic absorption peaks of the material (1400 nm, 1900 nm, and 2260 nm) become more pronounced.

Absorption and scattering properties of the aerogels can be observed by comparing the transmission (Figure 2) and reflection (Figure 3) spectra: τ(λ) diminishes for wavelengths less than 1400 nm, due to the material’s nano-pores, with a consequent proportional increase in ρ(λ), according to the literature [29,30]. Pores on the scale of the wavelength of the incident light are present in aerogels, and these pores can act as scattering centers. In previous studies, Zhao et al. [29] and Hunt [30] showed a wavelength dependence of scattered light that varies as 1/λ^4^, qualitatively consistent with the measured reflectance in the visible range. As the amount of MTMS in the recipe is increased, the spectral reflection increases (Figure 3). The effect becomes more pronounced from 7.5% MTMS to 10% MTMS. Because of the high values of ρ(λ) measured in the 380–500 nm range (peak value of 0.53 (HY 0)–0.83 (HY 10) at 400 nm), the light reflected by the aerogel appears bluish. For the whitish HY 10 pane, reflectance before and after aging spectra overlap in the NIR range, whereas a reduction of about 10% is registered in the visible range. For HY 7.5 and HY 0, the reflectance after aging increases by a moderate amount (in the 0.6%–4.2% range). The translation to the right confirms that reflected light moves away from the bluish tones of the new material towards a warmer shade, so the surface of the aerogel appears yellowish.

Light and direct solar transmittance (τ_v_ and τ_e_) and reflectance (ρ_v_ and ρ_e_) factors were calculated in compliance with EN 410 [24] and are shown in Table 1. Hydrophilic aerogel HY 0 has high transmittance for radiation in the solar spectrum, as well as in the visible part of the spectrum, with a behavior similar to conventional clear glass of 6 mm thickness [31]: values of τ_v_ and τ_e_ equal to 0.67 and 0.74, respectively, were found for the HY 0 sample before aging. The measured visible transmittance of HY 0 (τ_v_ =0.67) is similar to the value found in [32] (τ_v_ = 0.65) for a sample 90 kg/m^3^ dense and 12 mm thick. The hydrophobic samples HY 7.5 and HY 10 before aging show τ_v_ values of 0.60 and 0.37, respectively, and τ_e_ values of 0.70 and 0.57, respectively. A reduction in light transmittance of about 45% and direct solar transmittance of about 23% is found for HY 10 with respect to HY 0. The reflectance is consistent with the measured spectral transmittance, as expected: a relevant percentage of light is transmitted within the environment by the non-hydrophobic sample, for which, on the other hand, the light reflection is very low (ρ_v_ = 0.13). As hydrophobicity increases, ρ_v_ reaches values up to 0.41. Similar behavior is observed in the solar spectrum.

After an aging period equivalent to 12 years of natural service life, a deterioration in transmission properties occurs for HY 0, with a reduction in both light and solar transmission coefficients of 0.03 (reduction of about 4%). Furthermore, the visible reflectance increases by about 18% and the solar reflectance by 10%. For the most hydrophobic sample, HY 10, a reduction by 0.05 and 0.04 is observed for τ_v_ and τ_e_, respectively, corresponding to a 13% and a 4% change, whereas no significant changes are found in the reflectance coefficient. It results from higher radiation absorption by the material, consistent with the peaks observed in Figure 2.

A less significant influence of the aging process is observed for the sample HY 7.5, with a reduction of the optical parameters in the 0.02–0.03 range (3–4% less in terms of transmittance).

The prolonged aging of up to 12 years results in a yellowing of aerogel’s surface, related to the right shift of the reflectance spectra. This behavior was not perceptible after 2.5 years of aging when the reflection spectra of the new and aged material still appeared to overlap [20]. However, it is a very influential aspect for future improvements of the aerogel because transparency is a prerogative for a glazing element, with typical service life around 20 years.

### 3.2. Acoustic Properties

The as-prepared samples were used to estimate density. The measured density values are 0.093 ± 0.005 g/mL, 0.091 ± 0.004 g/mL, and 0.092 ± 0.004 g/mL for HY 0, HY 7.5, and HY 10 samples, respectively. Density measurements were not repeated after aging because some samples were broken, which made the volume difficult to evaluate.

**Figure 4:**
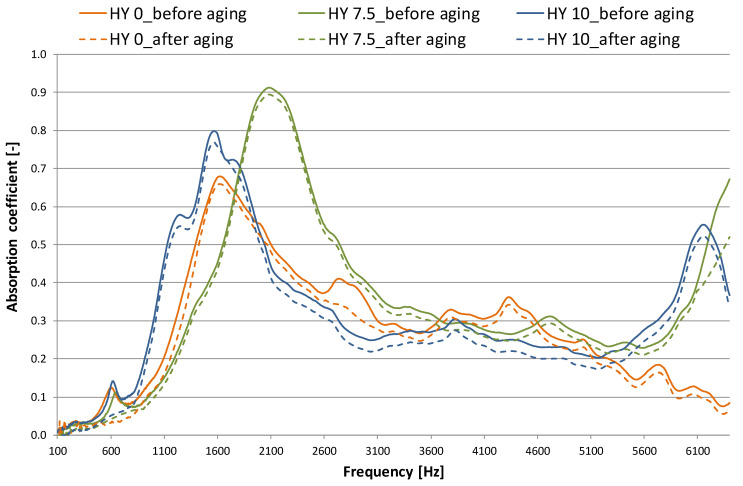
Normal incidence absorption coefficient for monolithic aerogel panes (large and small tube combination), influence of sample hydrophobicity: performance before and after 12 years of aging.

**Figure 5:**
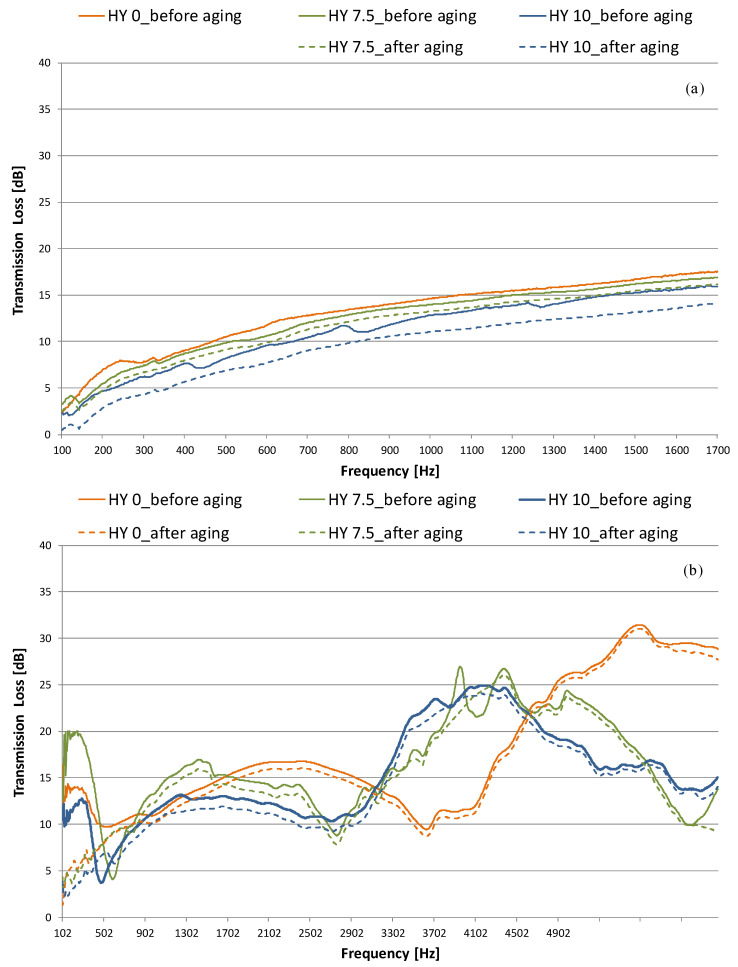
Normal incidence transmission loss for monolithic aerogel panes: influence of sample hydrophobicity. Performance before and after 12 years of aging: (**a**) large tube; (**b**) small tube.

Figure 4 and Figure 5 plot normal incidence absorption coefficient (α) and Transmission Loss (TL) results, respectively, comparing the behavior before and after aging [25,27]. Measurements were repeated at least three times on each specimen by rotating it inside the tube, and the results were averaged. The absorption coefficient (Figure 4) increases with hydrophobicity, and given the comparable density of the HY 0, HY 7.5 and HY 10 samples, this is likely due to structural differences. All the samples show a peak at low frequencies (less than 2500 Hz), whereas lower absorption occurs at medium frequencies. For the hydrophobic samples, a new peak occurs above 5000 Hz. The absorption coefficient peak increases as sample hydrophobicity increases, but the frequency of the peak value seems to be independent of hydrophobicity itself (α ≃ 0.68 at 1600 Hz for HY 0, α ≃ 0.80 at 1500 Hz for HY 10, α ≃ 0.91 at 2100 Hz for HY 7.5 sample).

After aging for 12 years, the HY 7.5 sample shows the least change in absorption coefficient. For all the specimens, a slight absorption peak reduction is observed (from 0.68 to 0.66 for HY 0, from 0.91 to 0.89 for HY 7.5, and from 0.80 to 0.77 for HY 10). The hydrophobic aerogels (HY 7.5 and HY 10) at the frequencies with the maximum absorption coefficient (in the 1600–2600 Hz range for HY 7.5 and the 1100–2100 Hz range for HY 10) retain almost unchanged properties compared to the situation before aging. HY 0 has a further lowering in the 2600–3100 Hz range.

Transmission loss levels are plotted in Figure 5a,b. The large hydrophilic sample broke, so it was impossible to measure the transmission loss value after aging. In general, the acoustic insulation properties of all the samples were not very good. Data measured in the 100–1600 Hz range (large tube) show that when the hydrophobicity increases, the TL value decreases (0.7–2.5 dB). Moreover, transmission loss increases as the frequency increases for the same hydrophobicity, in agreement with the mass–frequency law. Measurements performed in the small tube confirmed this trend up to 3300 Hz. For higher values, the trend is reversed, and the less hydrophobic samples have smaller transmission loss. The HY 0 specimen shows a drop at about 3300 Hz.

Acoustic insulation is relatively high, especially for the HY0 monolithic aerogel, which shows a TL of 17.5 dB at 1600 Hz (100 mm-diameter sample), whereas it increases above 1600 Hz, with peak values in the 20–27 dB range at about 4500–4900 Hz.

For similar thicknesses, as found in previous work [33], the maximum transmission values are obtained for higher frequencies, and the trends are similar for the samples of differing hydrophobicity.

The aging results for large samples (Figure 5a) show a slight decrease in TL with increasing hydrophobicity. Small tube analysis on 28 mm samples (Figure 5b) highlights a significant reduction of the peak for HY 7.5 at 3900 Hz and the one for HY 10 at 3700 Hz after 12 years. Anomalous behavior is observed below 500 Hz for the samples before aging, probably due to problems with the instrument at low frequencies.

In order to compare the samples, the noise reduction coefficient (NRC) was calculated as a mathematical average of the absorption coefficient values in the one-octave band at frequencies of 250, 500, 1000, and 2000 Hz, rounded to the nearest 0.05 (Table 2 and Table 3). This quantitative index is not specifically relevant to data acquired from impedance tube tests; nevertheless, a similar approach has been previously followed by others [34,35]. A noise reduction coefficient of 0.21 was found for the hydrophilic sample HY0, and it increased when hydrophobicity increased: values of 0.23 and 0.25 were found for HY 7.5 and HY 10, respectively.

The results of the samples before aging are in agreement with those of previous work in which monolithic and granular aerogel acoustic performance were investigated. Values of NRC in the 0.20–0.22 range were found for monoliths [33], whereas slightly higher values were found for the granular form (NRC in the 0.23–0.27 range, depending on the granule size) [34]. Aging affected the noise reduction coefficient (NRC) of all the samples, with a reduction in the 0.02–0.03 range (Table 2) primarily due to the decrease of α in the octave band at 500 and 100 Hz (Table 3). In particular, the percentage of reduction decreases with the frequency: it is only 3–6% at 2000 Hz and it is variable in the 30–60% range at low frequencies (250–500 Hz).

### 3.3. Color Rendering

In the Supplementary Materials section, Figure S1 shows the color shift, ΔE, values of the 24 color patches for each sample before and after the aging process. In general, ΔE values increase when hydrophobicity increases (see the ΔE ranges in Table 4).

The HY 10 sample had the worst behavior (i.e., largest color shift) in terms of color rendering even before the aging process.

All samples before and after aging showed the highest color shifts for colors B1, B6, C3, and C4, which are the yellow–orange–red tones. It was observed that HY 0 and HY 7.5 showed a further worsening of the color rendering after aging, with a further increase in ΔE; moreover, HY 10, with ΔE values always higher than HY 0 and HY 7.5, does not increase ΔE after aging, but is the same or slightly lower.

All samples show the lowest ΔE values for colors A6, D1, D2, D3, and D4, which are the light blue–gray tones. The ΔE variations before and after aging are very low and do not show an obvious trend.

**Figure 6:**
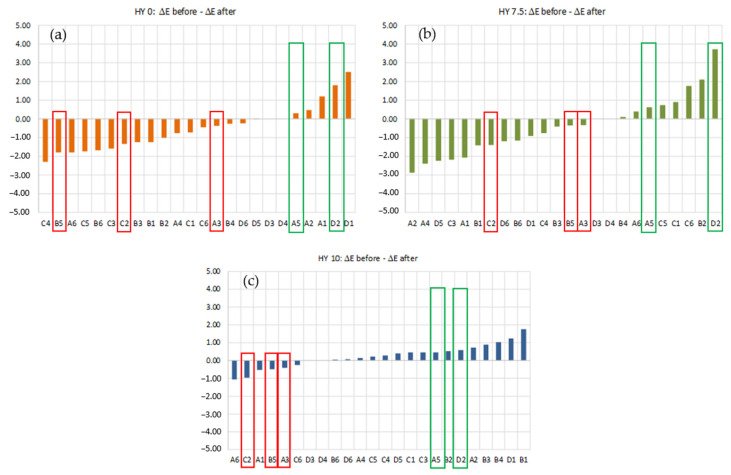
Differences between color shift values before and after aging of each hydrophobicity: (**a**) HY 0; (**b**) HY 7.5; (**c**) HY 10.

The differences between the color shifts before and after aging are plotted in Figure 6 for each sample. Negative values indicate a deterioration in color rendering, whereas the positive ones indicate an improvement. Aging has little effect on the color rendering of the more hydrophobic aerogel HY 10: the ΔE variation before and after aging is in the −1 to + 2 range (Figure 6c), which is negligible. This is confirmed by the color rendering index R_a_, which is the same (equal to 61) even after the aging process (Table 5). HY 7.5 shows the most significant influence of aging, with the ΔE variation before and after aging in the −3 to +4 range (Figure 6b). In this case, the R_a_ value decreases from 89 before aging up to 75 after aging, a reduction of about 16% (Table 5). An intermediate behavior is observed for HY 0: the ΔE variation before and after aging is in the −2.5 to +2.5 range (Figure 6a), and R_a_ value decreases from 84 to 74, or about 12% (Table 5). It can be observed that the extent of hydrophobicity does not influence the aging behavior so much in terms of color rendering properties.

Figure 6 shows that for all samples, after aging, colors A3, B5, and C2 have decreased ΔE and colors A5 and D2 have increased ΔE. The ΔE variations before and after aging are highlighted in red (worsening) and green (improving) boxes in Figure 6. Table S1 in the Supplementary Materials presents the RGB coordinates for each color.

The deterioration after aging involves light-green shades B5 and C2 and azure A3, which show the highest G- and lowest B-coordinates. On the other hand, the D2 and A5 hues characterized by both high G- and B-coordinates improve for the aged aerogels for every hydrophobicity (Table S1). In general, the R- and G-coordinates increase about 1–4%, and the B-coordinate reaches an increase of 6–7% for A3 and C2. The variation in the B-coordinate percentage becomes more negligible as the hydrophobicity increases. The specimen loses transparency, becoming more whitish, so the colors with a more significant presence of blue move away from their original hue to tend toward white.

### 3.4. Physical Properties

Table 6 presents the results of the BET surface area and BJH pore distribution analyses for a single as-prepared and aged aerogel sample at each MTMS level (HY 0, HY 7.5, and HY 10). The surface area increases as the hydrophobicity increases, in agreement with previous results [36]. The peak pore size (the pore size with the greatest pore volume) is about 20–30 nm. The results indicate that the aged samples have a high surface area (and are still aerogels). However, it is impossible to determine if the differences between the as-prepared and aged samples are meaningful. A low number of samples was tested, and it was not possible to analyze the same sample before and after aging due to the destructive nature of the BET/BJH tests. The standard deviation characterizes measurement uncertainty, not sample-to-sample uncertainty.

**Figure 7:**
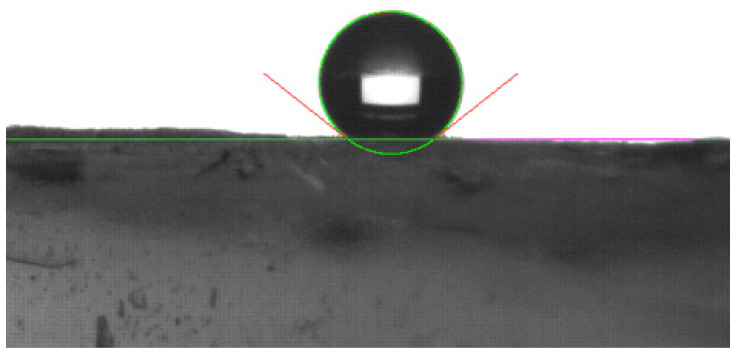
Image of a 2 µL drop of deionized water on the surface of an HY 10 silica aerogel monolith. Contact angle measured to be 142.3° at left and 141.8° at right.

Prior to aging, the HY 7.5 (2 samples, 10 locations each) had a contact angle of 125° ± 3°, and the HY 10 (3 samples, 10 locations each) had a contact angle of 141° ± 4°. A representative image is shown in Figure 7. After aging, the aerogels were no longer hydrophobic: water drops on the surface of the HY 7.5 and HY 10 monoliths were immediately absorbed. Further testing demonstrated that the interior of the monolith was also hydrophilic, indicating that hydrophobicity was lost throughout the aerogel matrix.

**Figure 8:**
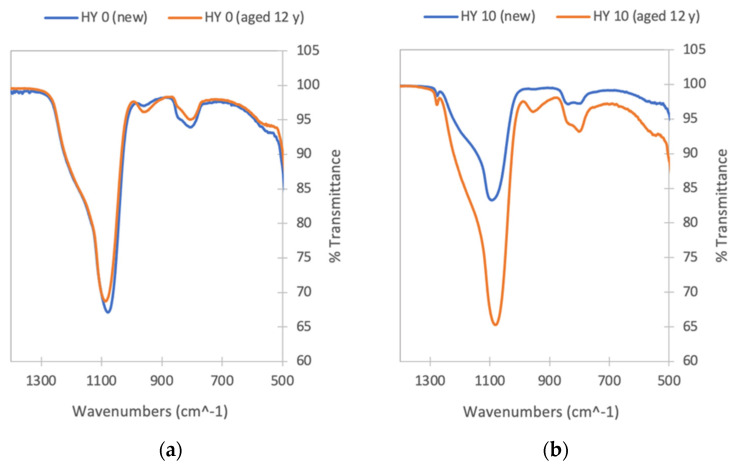
Infrared spectra of (**a**) HY 0 and (**b**) HY 10 silica aerogel as prepared (blue line) and following accelerated aging equivalent to 12 years (orange line). Note: the size of the peaks observed for different samples is impacted by the quality of the contact between the sample and the ATR crystal and is not necessarily representative of concentration differences between the samples.

FTIR analysis was performed on powdered aerogel samples to demonstrate whether the hydrophobic (Si-CH_3_) groups from MTMS are retained in the aerogels upon fabrication and upon aging. The spectra for HY 0 and HY 10 shown in Figure 8 emphasize the spectral region in which Si-O and Si-C vibrations can be observed. The spectra of as-prepared aerogels (prior to aging) are consistent with those obtained previously for aerogels prepared via the RSCE method using TMOS alone and with higher proportions of MTMS [36]. The major peak around 1100 cm^−1^, with a shoulder at higher frequencies, is due to Si-O-Si stretching [36,37,38] and is seen in all spectra. The sharp peak at 1276 cm^−1^ in the HY 10 spectrum (Figure 8b) is due to Si-CH_3_ stretching [39] and is thus not observed for the HY 0 samples. The same peak, with smaller intensity, is observed in the spectra of the HY 7.5 samples (not shown). Small peaks due to (Si)CH_3_ stretching (not shown) are also observed at higher frequencies in the spectra of the hydrophobic samples. Another peak of Si-CH_3_ is expected to be observed in the 760–865 cm^−1^ range [39]; however, the hydrophilic aerogel also has peaks in this region.

The peak due to the Si-CH_3_ bond vibration at 1276 cm^−1^ is present after aging (Figure 8b), indicating that the aerogel matrix still contains a significant number of the hydrophobic -CH_3_ groups. The spectra of the HY 7.5 and HY 10 aerogels after 12-year aging are otherwise remarkably similar to that of the HY 0 sample. This, in combination with the observed deterioration in hydrophobicity (from the sessile drop tests) provides indirect evidence that a change in structural (morphological) hydrophobicity, rather than simply a chemical decomposition, occurred during aging under the experimental conditions.

## 4. Conclusions

Monolithic aerogel is a promising material for the transparent building envelope due to its good thermal, acoustic, and optical properties. Color rendering characteristics are also of interest in interior lighting. However, the long-term performance of aerogel material has yet to be determined, and its behavior during a standard window life of about 20 years is worth investigating. Aerogel hydrophobicity and its effect on long-term performance is another characteristic of interest. Here, hydrophilic and hydrophobic silica aerogels were characterized in terms of hydrophobicity, porosity, optical and acoustic properties, and color rendering before and after an accelerated aging process. Artificial aging was carried out in the laboratory to reach an equivalent of 12 years, considered a significant step for progressive deterioration assessment.

Results of the measurements carried out before and after aging showed the following:

- high visible transmittance (peak of 0.89 for HY 0) is obtained. The value decreases slightly for HY 7.5 and up to 20% for HY 10 (τ_v_= 0.67, 0.60, 0.37 for HY 0, HY 7.5, and HY 10, respectively). On the contrary, reflectance increases as hydrophobicity increases, especially in the visible range. The aging process results in small but significant changes in the transmittance and reflectance properties;
- when the hydrophobicity increases, a moderate reduction in sound insulation performance (about 1–3 dB) and an improvement in absorption properties (NRC are in the 0.21–0.25 range) are measured. After aging, NRC- and TL-values are reduced up to 0.03 and 2–5 dB for the 10% MTMS sample;
- color shift increases with hydrophobicity. The highest values are obtained for yellow–orange–red tones. The aging worsens the color rendering with HY 0 and HY 7.5 panes (color rendering index R_a_ decreases by 10 and 14 for HY 0 and HY 7.5, respectively), whereas it is negligible with HY 10 (R_a_ = 61 before and after aging). The ΔE variations before and after aging are very low in light blue–gray tones. On the contrary, the aging process has a negative effect on light green and azure tones;
- the increase in the hydrophobicity of the monolithic silica aerogel involves an increase in surface area (reaching 640 m^2^/g). For hydrophobic samples, the contact angle increases with the amount of hydrophobic precursor employed in aerogel preparation, from 125° for the samples made with 7.5% to 141° for samples with 10% MTMS. Due to the hydrophobicity loss of the samples after aging, the contact angle tests are partially supported by a chemical composition analysis (FTIR). Some change in structure is observed, although the initial chemical composition is not entirely compromised: Si-CH_3_ groups are still present in the aged HY 7.5 and HY 10 samples.

To assess material changes after 20 years, the service life of most building elements, the samples will be subjected to a second artificial accelerated aging campaign to repeat the characterization and establish a performance trend of monolithic aerogel.

It is important to note that these aerogel samples are being tested outside of a glazing system, which is a “worst case” scenario. Further studies will be done to determine how monolithic aerogel ages when incorporated within a glazing system between panes of glass that would attenuate the exposure of the aerogel material to certain wavelength regions of solar light and to the environment (including humidity) of the unit.

## Figures and Tables

**Table 1:** Optical parameters calculated for the samples before and after 12 years of aging.

	Before				After							
Sample	τ_v_	τ_e_	ρ_v_	ρ_e_	τ_v_	τ_e_	ρ_v_	ρ_e_	∆τ_v_	∆τ_e_	∆ρ_v_	∆ρ_e_
HY 0	0.67	0.74	0.13	0.11	0.64	0.71	0.16	0.12	−0.03	−0.03	0.03	0.01
HY 7.5	0.60	0.70	0.16	0.12	0.58	0.67	0.18	0.14	−0.02	−0.03	0.02	0.02
HY 10	0.37	0.57	0.41	0.28	0.32	0.53	0.41	0.27	−0.05	−0.04	0.00	−0.01

**Table 2:** Noise reduction coefficient calculated for the tested samples before and after 12 years of aging and comparison with previously studied monolithic [33] and granular [34] aerogel samples.

Sample	NRC Value before Aging	NRC Value after 12 Years Aging
Granular (15 mm, φ = 0.01–1.2 mm) [34]	0.27	-
Granular (15 mm, φ = 0.7–2.0 mm) [34]	0.23	-
Monolithic (12.7 mm) [33]	0.20	-
Monolithic (19.1 mm) [33]	0.21	-
Monolithic (25.4 mm) [33]	0.22	-
Monolithic (29 mm) HY 0	0.21	0.18
Hydrophobic Monolith (29 mm) HY 7	0.23	0.21
Hydrophobic Monolith (29 mm) HY 10	0.25	0.22

**Table 3:** Absorption coefficient at octave bands for NRC calculation.

Octave Band	α_before_			α_after_		
	HY 0	HY 7.5	HY 10	HY 0	HY 7.5	HY 10
250	0.03	0.03	0.03	0.02	0.01	0.01
500	0.08	0.06	0.08	0.03	0.03	0.04
1000	0.23	0.16	0.37	0.18	0.15	0.34
2000	0.51	0.66	0.51	0.48	0.64	0.48

**Table 4:** TL ranges (dB) of the samples before and after aging.

Sample	Before	After
HY 0	10.2–59.1	8.4–60.7
HY 7.5	11.5–64.8	7.7–67.0
HY 10	12.1–84.0	11.6–83.7

**Table 5:** Color rendering index calculated with the new methodology proposed in [19].

Sample	R_a_		
	Before Aging	After Aging	Variation
HY 0	84	74	−10
HY 7.5	89	75	−14
HY 10	61	61	0

**Table 6:** Surface area and pore distribution of different hydrophobicity aerogel samples before and after aging.

Sample	Before Aging			
	Surface Area (m^2^/g)	Peak Pore Size (nm)	Surface Area (m^2^/g)	Peak Pore Size (nm)
HY 0	460 ± 10	20–23	--	--
HY 7.5	600 ± 10	25–28	680 ± 20	27–29
HY 10	640 ± 20	20–23	570 ± 10	32–34

## Data Availability

Further data are available on request from the corresponding author. For the sake of brevity, some data are not publicly available.

## Supplementary Materials

The following supporting information can be downloaded at https://www.mdpi.com/article/10.3390/gels9030210/s1. Figure S1: Comparison between color rendering variation of each aerogel specimen before and after aging; Table S1: RGB coordinates for each color patch before and after the aging of the panes.
