# Supplementary material for: Long-Term Performance of Monolithic Silica Aerogel with Different Hydrophobicities: Physical and Color Rendering Properties after an Accelerated Aging Process
Source: Gels. 2023 Mar 10;9(3):210. doi: 10.3390/gels9030210 (PMC10047942; doi:10.3390/gels9030210)
Supplement: Supplementary file 1 [file gels-09-00210-s001.zip › gels-2250052-supplementary.pdf]

Supplementary Materials

Long-term performance of monolithic silica aerogel with different hydrophobicities: physical and color rendering properties after an accelerated aging process

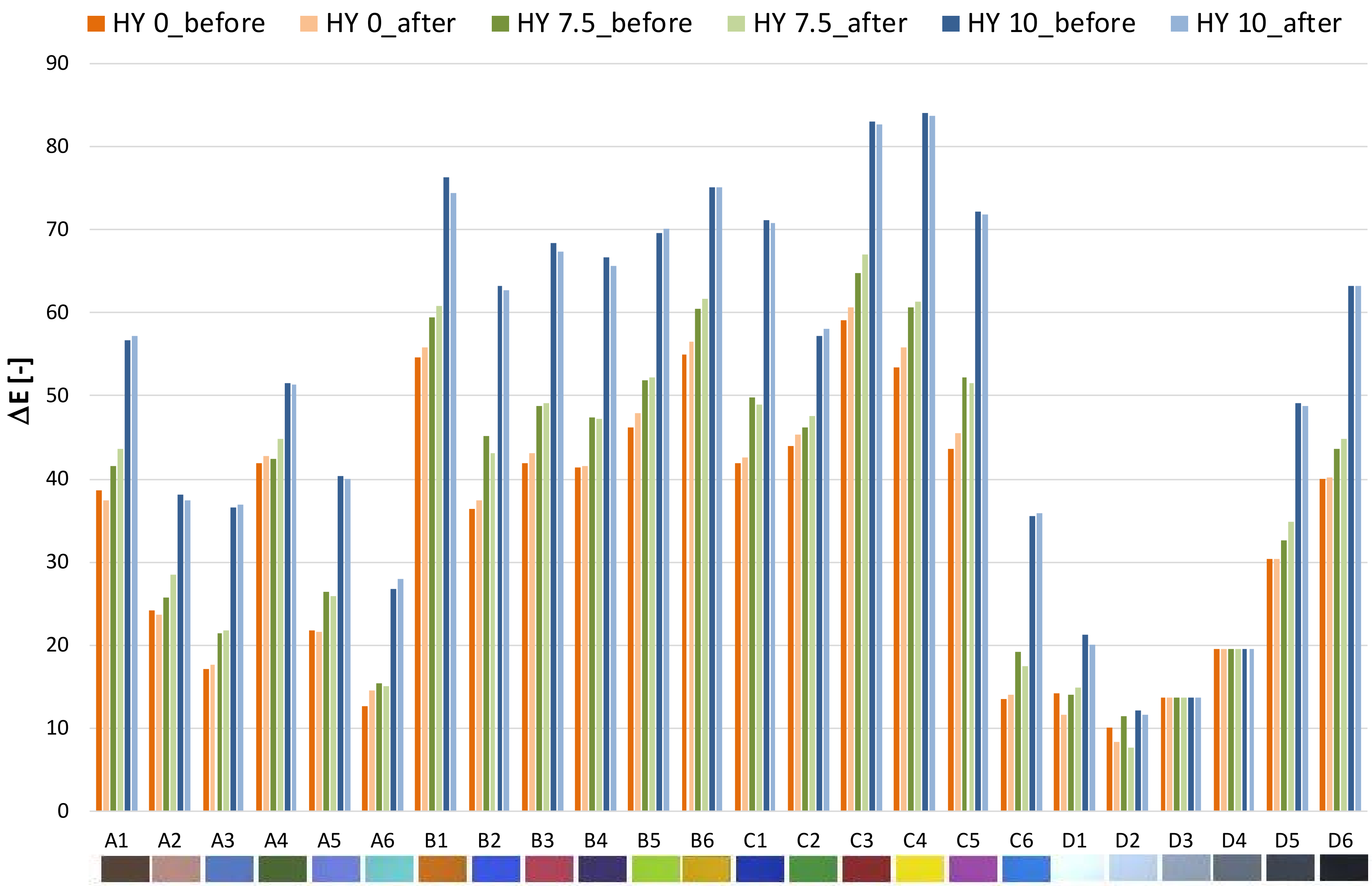

Figure S1. Comparison between color rendering variation of each aerogel specimen before and after aging.

Table S1. RGB coordinates for each color patch before and after the aging of the panes.

	R						G						B					
	HY 0		HY 7.5		HY 10		HY 0		HY 7.5		HY 10		HY 0		HY 7.5		HY 10	
	b*	a*	b*	a*	b*	a*	b*	a*	b*	a*	b*	a*	b*	a*	b*	a*	b*	a*
A1	114	115	127	126	172	169	169	167	189	190	255	255	196	194	208	216	255	255
A2	171	167	171	175	199	202	202	196	208	214	255	255	204	200	206	219	254	255
A3	118	122	132	131	176	180	194	196	206	210	255	255	228	230	222	238	255	254
A4	113	116	126	126	173	178	186	189	199	202	255	255	200	204	208	217	255	254
A5	131	133	139	141	182	186	195	197	204	210	255	255	232	234	220	240	254	254
A6	132	133	140	141	178	181	234	232	228	237	255	255	221	222	211	227	254	254
B1	178	179	176	181	196	202	187	190	199	203	255	255	176	181	190	199	255	255
B2	107	111	122	122	171	173	180	184	196	200	255	255	235	237	223	242	255	255
B3	169	170	169	174	199	203	174	177	191	193	255	255	200	204	205	217	255	255
B4	110	113	123	123	172	179	171	173	190	191	255	255	211	212	213	224	255	254
B5	158	158	161	163	197	201	242	239	239	245	255	255	182	184	195	201	255	255
B6	192	192	190	193	213	216	219	217	222	226	255	255	173	175	189	194	255	255
C1	96	99	111	111	165	165	169	171	185	190	255	255	219	218	212	228	255	255
C2	115	118	126	129	171	176	210	211	215	224	255	255	194	198	202	214	255	255
C3	148	151	153	158	190	193	167	173	187	192	255	255	197	201	205	218	255	255
C4	218	217	210	216	237	237	253	250	250	255	255	255	165	169	181	188	255	254
C5	162	162	164	167	197	199	175	178	192	194	255	255	217	219	214	227	255	255
C6	105	107	119	119	168	169	195	195	203	207	255	255	235	235	224	241	255	255
D1	229	255	221	229	246	242	255	255	255	255	255	255	248	242	216	252	255	255
D2	187	186	184	190	220	218	254	249	250	255	255	255	229	229	210	233	255	255
D3	155	156	158	161	196	199	226	224	227	235	255	255	219	219	213	229	255	255
D4	128	131	137	139	179	183	196	197	206	211	255	255	211	211	211	223	255	255
D5	108	111	122	122	168	173	176	178	192	195	255	255	204	205	210	221	255	255
D6	93	96	110	109	160	164	159	161	180	180	254	255	197	198	206	214	255	255

*b=before aging; a= after aging
